# Does the Prevalence of Ossified Fabella Vary in Knee Osteoarthritis and Age-Related Degeneration? A Meta-Analysis of About 11,000 Knees

**DOI:** 10.7759/cureus.12535

**Published:** 2021-01-06

**Authors:** Adil Asghar, Shagufta Naaz, Ravi K Narayan, Anup Kumar

**Affiliations:** 1 Anatomy, All India Institute of Medical Sciences Patna, Patna, IND; 2 Anesthesiology, All India Institute of Medical Sciences Patna, Patna, IND; 3 Orthopaedics, All India Institute of Medical Sciences Patna, Patna, IND

**Keywords:** risk ratio, fabellar prevalence, knee joints, osteoarthritis, age-related degeneration

## Abstract

Objective: Osteoarthritis (OA) and age-related degeneration (ARD) are stimulants for the development of the fabella in the knee joint. This meta-analysis updates previous studies and reviews on the prevalence of the fabella in OA or ARD knee joints. In addition, it provides a quantitative estimation of the fabellar prevalence in knees having OA and ARD.

Methodology: Twenty studies containing data from 11,056 knee joints were utilized in the investigation, consisting of 6,819 knees of individuals with OA (including those with age greater than 40 years) and 4,237 knees of individuals without OA (including less than 40 years of age), respectively. Totally, 2,434 knees of the OA subjects had fabellae (including more than 40 years), while in the non-OA subjects, 844 had fabellae (including less than 40years). The odds ratio (OR) and relative risk (RR) were calculated.

Results: The fabellar prevalence was observed to be higher in OA knees, where the risk ratio of developing fabella was 2.55 (2.15-3.02). Compared with this, the risk ratio for the incidence of fabella in ARD knee was 1.71 (1.59-1.85). The bilateral occurrence of fabella was more common than unilateral. The risk of developing fabella in individuals aged less than 40-year was 0.59 which was 41% less than individuals aged more than 40 years. The risk ratio of developing fabella in co-exposure of ARD and OA was 1.84 [1.66, 2.04, 95% CI].

Conclusion: OA and ARD would increase the prevalence of ossified fabella by 84%, thus acting as stimulants or associations and risk factors for ossified fabella.

## Introduction

The term fabella is Latin, meaning little bean. This sesamoid bone is embedded in the lateral head of the gastrocnemius in the posterior aspect of the femur [1]. It presents as a small fibrocartilaginous nodule developing after 8 to 12 years of age. The anatomical location of the fabella is at the intersection of tensile stress in the complex structure of the postero-lateral part of the knee, and it acts as a static stabilizer by reorienting various forces. Fabella increases the efficiency of the gastrocnemius muscle. It provides a biomechanical advantage in knee flexion [2]. It makes the fourth compartment of the knee joint (femorofabellar) and prevents friction-induced damage [1].

The knee joint is most commonly affected by osteoarthritis (OA). In severe OA, knee arthroplasty is a usual procedure these days. Fracture dislocation of fabella was uncommon before the era of knee arthroplasty [3]. The implant of knee arthroplasty frequently causes impingement of fabella or fabellar syndrome, which is related to fabellar degeneration and OA changes and is a matter of concern in patients after total knee replacement [2]. OA and degenerative changes in the knees generally begin in the fourth decade of life. However, the symptomatic presentation is commonly observed in the next decade. OA and aging or age-related degeneration (ARD) after 40 years are considered as associations or exposures for the formation of ossified fabella [4]. OA causes wear and tear in the articular cartilage, meniscus pathology, and osteophytes formation. These changes take place in femorofabellar articulation and subsequent, fabellar enlargement or irregularity in shape due to osteophyte formations and subchondral sclerosis. ARD may lead to degeneration of the articular cartilage or menisci and cartilaginous fabella. Subsequently, the degenerated fabellar cartilage undergoes dystrophic calcification which is another stimulant for fabellar ossification. The pathogenesis of ossification and enlargement of fabella could be similar to the above mechanism [4]. The prevalence of fabella varies from 3.1% to 86.9% and changes with ethnicity and the methods of observation. Prevalence of fabella is higher in OA knees, as reported in preliminary findings of a few studies. A hypothesis was formulated for this study based on the above evidence that OA and ARD of knee joint have a positive association with the presence of fabella. A meta-analysis was conducted to assess the prevalence of fabella in OA and ARD knees of subjects with age greater than 40 years. The meta-analysis also tried to assess the sex-linked prevalence and the laterality in the prevalence of fabella.

## Materials and methods

Literature review

Available literature was explored from the electronic database of PubMed/Medline, European PMC, CINAHL, Embase, EBSCO, Scielo, Clinical Key, Up To Date, OVID search, Google Scholar, AUSPORT, and Cochrane library from June 2019 to November 2019. The search included Medical Subject Heading (MeSH) terms such as prevalence, incidence, fabella, sesamoid bone, osteoarthritis, OA, arthritis, aging, ARD, knee pain, and knee degeneration in different strings of combinations. The string of terms was included in Boolean search with ‘OR, AND, or NOT’, and speech marks such as ‘Fabella or Knee Sesamoid or Popliteal sesamoid’ and ‘Fabella or Prevalence or Osteoarthritis’ or ‘Fabella or Prevalence or Knee pain’, to acquire maximum relevant articles. The delimiter ‘NOT’ was used to obtain appropriate studies. The strategy used for the PubMed database is mentioned at the end of the manuscript. Appropriate published articles were collected from the journals of subjects like Anatomy, Anthropology, Orthopaedics, Sports Injuries, Biomechanics, Morphological Science, and Radiology from websites or library archives. These search strategies yielded 119 works.

Literature selection

These works were shortlisted based on titles and abstracts by Rayyan QCRI App for a systematic review. Case reports and case series were excluded because they did not provide sample sizes, making risk estimation impossible. The publications without useable data, or where data did not exhibit 95% confidence intervals, were also excluded from the analysis. Studies providing risk estimates of OA or ARD were included. Further, to ensure an unbiased approach in selecting the studies, a summary of conferences, preprint articles, and published articles were also included. In addition, email correspondences were performed to gather unpublished figures of published manuscripts. Case reports accompanied with literature review were included for references, but their data were excluded from the analysis. The average prevalence from course-books or published manuscript without sample references were neglected. No restriction was applied based on the year of publication, language, or ethnicity. Studies reporting fabella before 12 years of age in a population sample were not considered because the development of fabella in this age group could not be confirmed. In addition, if an author confused popliteal artery calcification with fabella in radiographic findings, the results were excluded as they were not appropriate for analysis. The radiological studies incorporated the data of radiographs, CT, and MRI scans. The figures of USG and PET scans were omitted from the analysis because of their low detection rates. Anatomical studies reporting the age of cadavers and OA changes were included in the analysis.

The prevalence of fabella was measured in knees of OA subjects, non-OA subjects, subjects aged less than 40 years, and subjects aged more than 40 years. The Osteoarthritis Initiative (a project of the National Institute of Health) recommended the lowest age of 45 years for the OA cohort study, with a baseline of five years of exposure. Consequently, 40 years of exposure was considered as a baseline for ARD in this analysis. The prevalence of fabella was measured separately in males and females. In addition, the incidences of unilateral or bilateral fabella, and right or left were noted. The data on the size of fabella were not used because of insufficient sample size and variable methods of measurement.

The primary outcome was to measure the risk estimates of fabella in ARD and OA knee based on radiological and anatomical assessment. The assessment of the prevalence of fabella based on sex, laterality, and ancestry were secondary outcomes.

Assessment of risk of bias

Evaluation and analysis for the risk of bias in all selected articles were performed by using the Anatomical Quality Assurance (AQUA) tool of the International Evidence-Based Anatomy (iEBA-WG) working group. Two authors assessed the risk of bias independently. Any disagreement was resolved by a third author. The risk of bias was evaluated in all five domains - objective and subject characteristics, study design, methodology characteristics, descriptive anatomy, and reporting of results. Additionally, ROBINS I was utilized to assess the integrity or quality of these observational studies because the AQUA tool was inadequate to evaluate the exposure (intervention) in them.

Data extraction and statistical analysis 

Authors independently extracted the data and information relevant to the study using a standard data extraction form. The form included the following details: the number of participants, age, sex, inclusion, and exclusion criteria. Also, the statistics recorded were the publishing year, geographical area or population, the modality of study (anatomical or radiological), the number of individual knees appraised, and the number of fabellae detected (events). The contingency tables were prepared, and the odds ratio (OR), as well as 95% confidence interval, were computed. The risk estimations for age, gender, and laterality were investigated individually. No prior data were accessible to adjust these confounding factors. The Kellgren-Lawrence radiological grade was used to assess the OA in two radiological studies. Mean K-L score were computed in knees having fabella and without fabella. Fabella degeneration grade (FDG) was examined: grade 0 for normal fabella which having a triangular or oval shape and smooth articulating surface, grade 1 articular surface subchondral sclerosis; grade 2 sclerotic fabella with osteophyte formation at the fabellar margins; and grade 3, enlarged fabella (over 2 cm) with large osteophyte formation [2].

The unit of analysis was the prevalence of fabella in 100 knees examined. ProMeta v3 - Idostatistics and Revman 5.3 were used to analyze the pooled data. The OR and effect size were measured for each included study. The heterogeneity value was measured as i2. If i2 was <50%, then the fixed effect model was implemented; else, the random effect model was utilized. The Cochrane Q statistics was investigated. The OR, RR, and risk difference (RD) were measured with a 95% confidence interval. Sensitivity and cumulative analysis were performed to test the consistency of the findings. Publication bias was measured by the funnel plot. For the funnel plot, the logarithmically transformed OR against the standard error was utilized. Regression analysis was also performed to investigate the correlation between outcome and moderators (age, gender, and ethnicity). The publication bias was visually examined in the funnel plot along with Egger’s linear regression test and Begg and Mazumdar’s rank correlation test. The file drawer effect was investigated by generating Rosenthal Fail-Safe Number (FSN). Subgroup analysis was done to measure the risk estimates related to intervention if heterogeneity was above 50% because of the distribution of ethnicity or population and mode of study. The extracted data were not suitable to study the dose-response and dose-time effects. Finally, the OR, RR, and RD were computed based on data in the random-effect model.

## Results

Description of studies 

**Figure 1:**
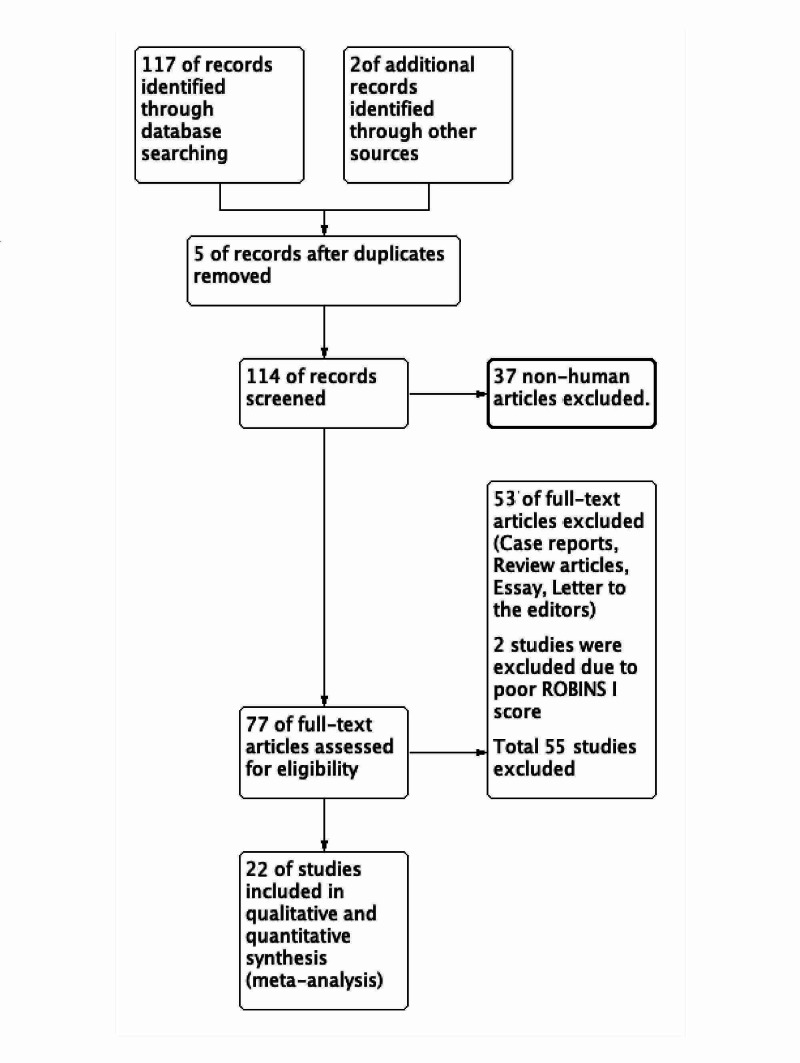
PRISMA flow chart.

The publications included were cross-sectional observational studies as case-controlled or cohort studies were unavailable despite extensive searches. One hundred seventeen relevant works were found as a result of electronic surveys, and two studies retrieved from conference proceedings. Five duplicated searched items were omitted. Another 37 studies were excluded because of non-human reports or case reports and series, review-based abstract, and title evaluation. Seventy-seven studies qualified after the title and abstract evaluation and three authors studied full texts of each work individually. A study was included based on the consensus of at least two authors. The interrater agreement was 0.87. For the meta-analysis, 22 studies from the years 1875 to 2019 were selected, which dealt with the prevalence of fabella associated with OA or ARD changes based on their abstract and full-text analysis. Of these, two studies [5,6] were excluded after evaluation of their risk of bias (ROBINS I) because of missing data. As a result, 20 observational studies [1-3,7-24] were included (Figure 1; Table 1).

**Table 1:** Characteristics of included studies. The characteristics of included studies and risk bias based on ROBINS I (for intervention-based observational studies) Radiological method includes radiographs, CT, and MRI (X-ray: radiograph, CT: computerized tomography, MRI: magnetic resonance imaging). The anatomical method includes the evaluation of the dissected knee. D: dissected specimen, CS: cross-sectional study, RS: retrospective study, PS: prospective study. ARD: Age-related degeneration (exposure of 40 years), OA: osteoarthritis. ^#^Ghimire et al. was removed from the analysis because of discrepancies in the data from published literature and graph. ^##^Data of exposure (Intervention) were presented in forest plots. *ROB: the risk of bias for intervention was evaluated by ROBINS I for all studies. L: low and M: moderate. The overall risk of bias was low to moderate.

Author	Year	Method	Study	Country	Exposure^##^	ROB^*^
Berthuambe and Bull [1]	2019	CT	RS	Korea	ARD	L
Hou et al. [2]	2019	X-ray	RS	China	OA	L
Hur et al. [3]	2020	X-ray	RS	Korea	OA	M
Gruber [7]	1875	D	CS	Russia	ARD	M
Hagihara et al. [8]	1993	D	CS	Japan	ARD	L
Sutro et al. [9]	1935	X-ray	RS	USA	ARD	L
Pritchett [10]	1984	X-ray	RS	Europe	OA	L
Ghimire et al. [11]^#^	2017	X-ray	CS	Nepal	ARD	M
Sonntag [12]	1930	X-ray	CS	Germany	ARD	M
Kitahara [13]	1935	X-ray	RS	Taiwan	ARD	L
Hessen [14]	1946	X-ray	RS	Sweden	ARD	L
Yano [15]	1928	D	CS	Japan	ARD	L
Chung [16]	1934	D	CS	Korea	ARD	M
Lungmass [17]	1954	X-ray	RS	Germany	ARD	L
Piyawinijwong et al. [18]	2012	D	CS	China	ARD	L
Tabira et al. [19]	2012	D	CS	Japan	ARD	L
Ehara [20]	2013	MRI	PS	Japan	ARD	L
Hauser et al. [21]	2015	D	CS	Europe	ARD	L
Egerci et al. [22]	2016	D	CS	Japan	ARD	L
Pop et al. [23]	2018	MRI	CS	Romania	ARD	L
Sohn et al. [24]	1985	X-ray	RS	Korea	ARD	L
Kato et al. [25]	2012	D	RS	Japan	ARD	M

Risk of bias in the included studies

The AQUA tool probed for potential risk of bias in five study domains, as previously mentioned. The risk of bias within each domain was categorized in percentage. A bias of less than 20% was considered low risk, 20-40% was categorized as moderate risk and >40% was high risk. The majority of the works included in this meta-analysis had a low to moderate risk of bias in domain 1 (objectives and subject characteristics) and domain 3 (methodology characterization) because of missing baseline demographic data of the study population and lack of information regarding the experience of the researchers. Almost all studies revealed a low risk of bias in the remaining domains (study design, descriptive anatomy, and reporting of results). The agreement varied from average to excellent. ROBINS I risk of bias was found as low to moderate in the 20 studies (Table 1). A reporting bias might be due to different methodologies. The cartilaginous fabella in the dissected knee may increase anatomical prevalence as only ossified fabellae were visible in radiographs. The inter-rater agreement between the two authors was 69.2 [53-78%].

Effect of exposures

Eleven thousand fifty-six knees were evaluated for risk estimates in co-exposures of OA and the ARD processes. The risk estimate for the prevalence of fabella in combined exposure had an OR of 2.16 (1.68, 2.76 95% CI), and a RR of 1.71 (1.40, 2.10 95% CI). The risk estimate of prevalence of fabella in persons <40 years of age had an OR of 0.54 (0.42, 0.71 95% CI) and RR of 0.59 (0.54, 0.66; 95%CI). All studies revealed a serious heterogeneity, and the heterogeneity statistics (i^2^) was 81%, while the Cochrane Q value was 96.38 for df = 19 (P = 0.0001). It was unacceptable (i^2^ acceptable up to < 50%) to combine the risk estimate, and needed subgroup analysis to deal with heterogeneity.

Subgroup analysis

Considering that either OA or ARD was producing heterogeneity, subgroups were created, with OA studies being shifted into the ‘OA’ subgroup and the remaining studies moved into the ‘ARD only’ subgroup. The ‘ARD’ subgroup still exhibited heterogeneity, and this was dealt with using the eyeball test. It was observed that Ghimire et al. [11] were producing heterogeneity. Upon further analysis, the data of Ghimire et al. were found faulty and were removed from the analysis, which reduced heterogeneity (i2) to 37%. After the removal of an outlier, heterogeneity was within the acceptable limit. The risk estimate for OA and ARD were measured again in 10,890 knees.

OR and RR for co-exposure were 2.42 [2.03, 2.87 95%CI] and 1.84 [1.66, 2.04 95%CI], respectively, which meant that the combined effect of ARD and OA increased the prevalence of fabella by 84%. Further sub-group analysis was done to elucidate the individual effects.

Effect of Age-Related Degeneration of Knee

**Figure 2:**
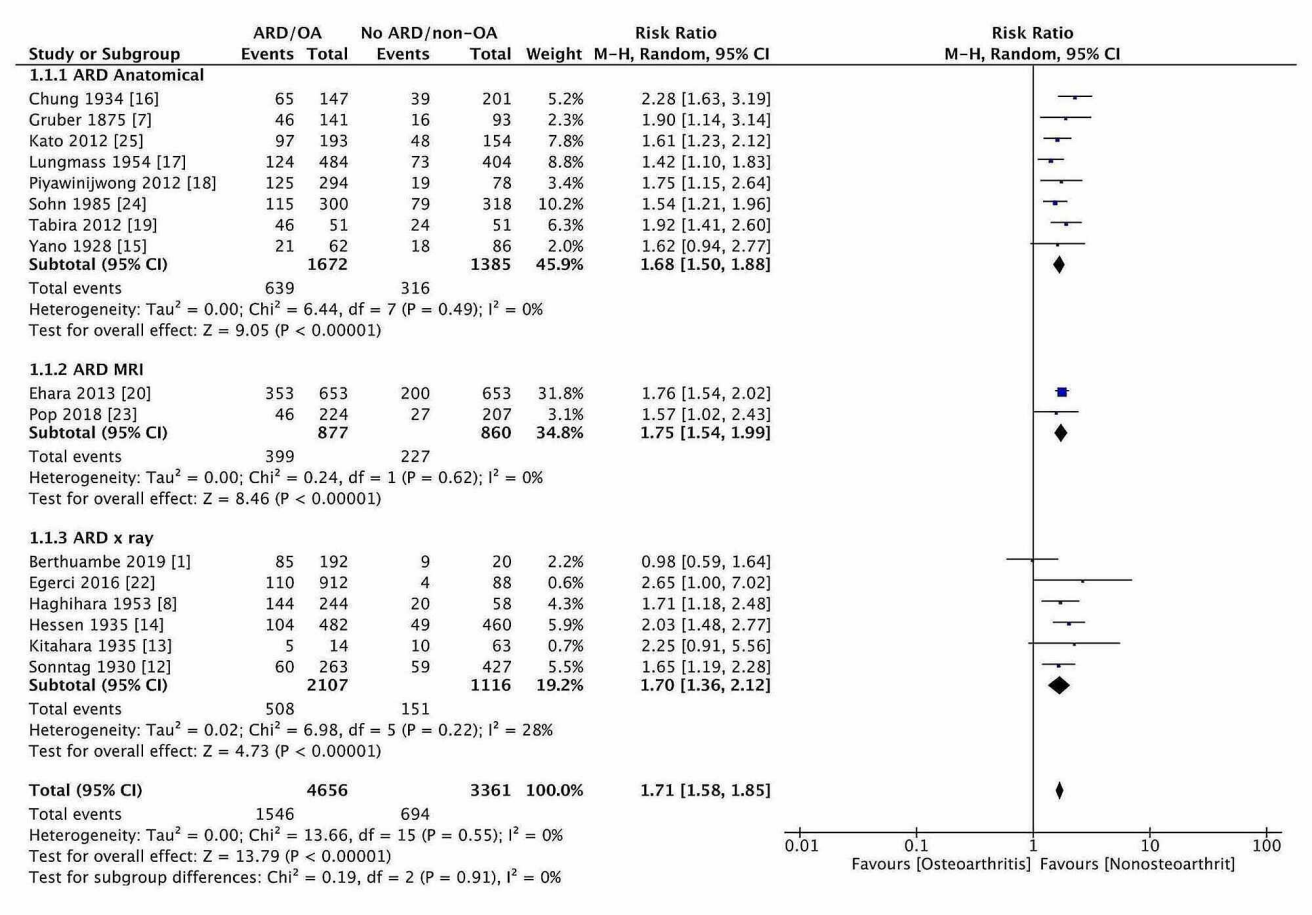
The forest plot of fabellar prevalence in ARD and along with subgroup analysis.

OR and RR for ‘ARD’ in 8,005 knees were 2.22 [1.89, 2.60 95%CI] and 1.71 [1.59, 1.85 95%CI], respectively, and ARD would increase the fabellar prevalence by 71% (Figure 2). The result was again sub-grouped according to the method of studies. The risk of developing fabella (RR) in more than 40 years was 1.68 (1.50,1.89, 95% CI), 1.75 (1.54, 1.99, 95% CI), and 1.70 (1.36, 2.12, 95% CI) for anatomical, X-ray/CT, and MRI studies, respectively. Overall, no significant variation in the risk estimation whatever, the study modalities adopted. The RR for developing fabella ‘under 40 years’ was 0.59 [0.54, 0.66 95% CI], which meant ARD increased the prevalence of fabella by 41%. The difference of 30% risk in age ‘more than 40 years’ might be due to the early development of OA, which was not recognized in radiographs. The fabellar degeneration with age was examined in a single reported study. The moderate association with age was present (r = 0.53 Spearman non-parametric correlation, p < 0.0001).

Effect of Osteoarthritis

**Figure 3:**
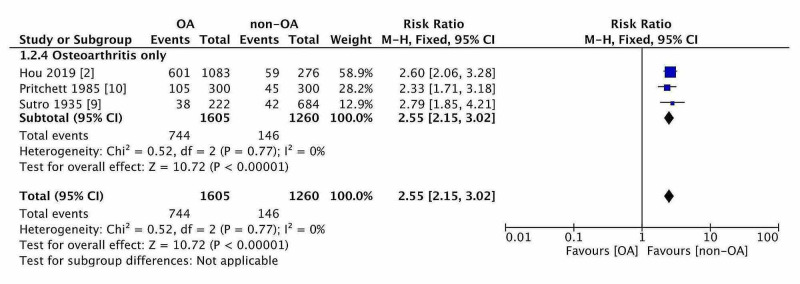
The forest plot of risk estimation for developing ossified fabella in osteoarthritis.

**Figure 4:**
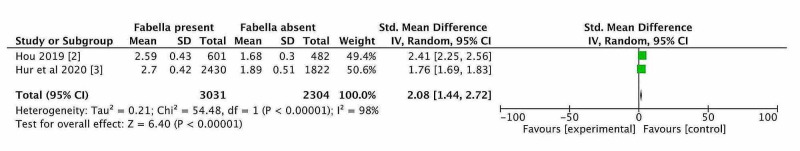
Forest plot of K-L grade score of knees with or without fabella.

The OR and RR for ‘OA’ in 2,865 knees were 3.81 [2.56, 5.68 95% CI] and 2.55 [2.15, 3.02 95%CI], respectively. Thus, OA had increased the prevalence of fabella by 155% (Figure 3). The baseline prevalence of fabella in the non-OA knee was 17-19% but the same OA knee was 51% (almost three times). Only two studies were available which discussed the prevalence of fabella according to Kallagren-Lawrence grading (K-L grade) of knee OA. The prevalence of fabella was measured in all five grades of knee OA (K-L grade 0-4). The mean and standard deviation of K-L grade were computed in both studies. Then, a standardized mean difference (SMD) of KL grade was computed for both studies when fabella present and fabella absent in OA knees. The pooled SMD of KL grade was 2.08 [1.44, 2.72, 95% CI] with significant heterogeneity due to population difference. The forest plot (Figure 4) was generated which showed that knees having fabella have a higher K-L grade score than knees without fabella. The degeneration of fabella was also studied in relation to the K-L grade score. The regression analysis was conducted between the K-L score and fabellar degeneration score in 1359 knees. The association was noted between the two (r = 0.99, p = 0.00095).

Effect of Gender, Laterality, and Ethnicity

**Figure 5:**
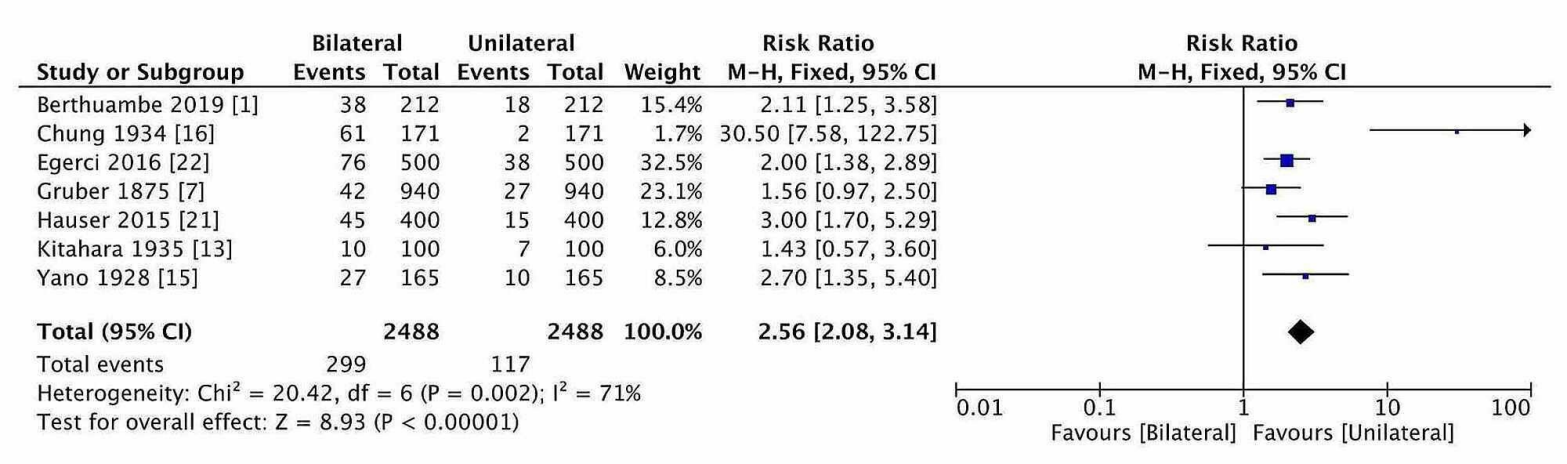
The forest plot of laterality distribution of fabella (bilateral vs unilateral).

Female participants had a 2% higher risk (RD) of the prevalence of fabella in comparison with males (P = 0.13). Laterality analysis did not show any RD of fabellar prevalence, and fabella was distributed equally on both sides (the diamond of risk estimate located on ‘no effect line’). The prevalence of bilateral fabella was higher than unilateral fabella, but estimation was done based on seven studies, and the estimated RR was 2.56 (2.08-3.14; 95% CI) in OA and ARD (Figure 5). Thus, bilateral fabellar prevalence was 156 % higher than the unilateral. The RR of fabella in the USA (North American) and Middle-East Asian were 2.65 and 2.18, but quite lower in European (1.79) and Asian Mongoloid ethnicity (1.65). The observed higher risk in North American or Middle-East Asians could be attributed to the smaller sample size.

The data pooled from Asian-Mongoloid ethnicity (Chinese, Japanese, Korean, and other nearby ethnicities) presented significant heterogeneity compared with European studies. Data from the USA and the Middle East could not be compared due to insufficient studies for comparison. There was no significant difference between the OR of studies from journal articles and conference presentations. The meta-regression of the effect size of fabellar prevalence with the year of publication had an insignificant association. Sensitivity and cumulative analysis were done to detect RR variation after adding and removing each study, respectively. The variation in RR was constant with minimum variation (0-0.05) after 4,768 knees were included in the studies. The authors examined approximately 11,000 knees, and RR will not vary beyond this range. Hence, it was determined that any further study would not impact the RR.

Publication bias

**Figure 6:**
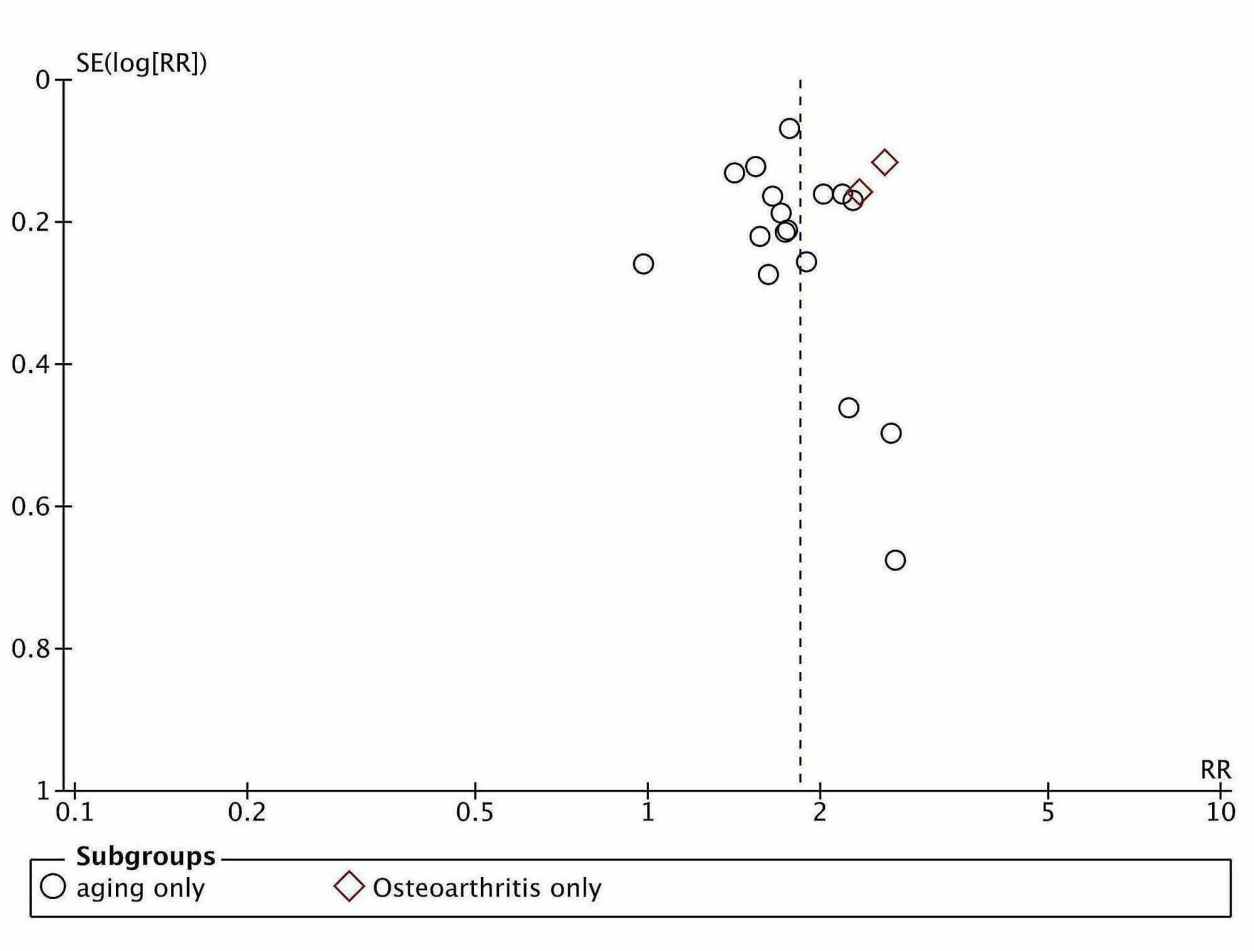
Publication bias of included studies.

The publication bias was guessed to be present, as exhibited in the funnel plot, but that might be due to sample variations (Figure 6). Egger’s regression test (P = 0.517) and Begg and Mazumdar’s rank correlation test (P = 0.846) confirmed no publication bias. Rosenthal’s fail-safe number was estimated and found to be higher than the Rosenthal rule of thumb 5k + 10 value, e.g., 110 (k = 20). Rosenthal's FSN passed the File drawer test. Reporting bias was expected in the qualitative analysis, but the quantitative analysis refuted the assumption. Thus, the observed prevalence did not need adjustment. Dose-response meta-analysis was not performed due to a lack of suitable data.

## Discussion

This meta-analysis summarizes the finding of 20 observational studies, encompassing a total of 10,890 knees, investigating the association of prevalence of fabella and OA along with ARD. This analysis presented the increased risk of developing fabella in an OA knee. The ARD process has an additional impact on the prevalence of fabella. This analysis measured the risk estimates of the cumulative as well as individual effects of OA and ARD. Sufficient data were unavailable to find age-adjusted risk estimates of fabellar prevalence in OA. The fabellar prevalence was higher bilaterally and on the right side in case of unilateral prevalence. A similar distribution was found in the literature for OA. OA has bilateral with asymmetrical presentation and farther on the right side in terms of severity, which mimics the distribution of fabella in OA [4]. In this meta-analysis, publication bias may have a minor role due to the existence of some unpublished data. The publication bias and other biases were reduced to a minimum by excluding studies that did not meet the selection criteria and ROBINS I grading. As very few small sample sized studies were missing on examining the funnel plot, so it did not affect the risk estimates. Some degree of heterogeneity was due to the differences between the methodologies and the study populations.

Potential biases in the study 

Because only 19 studies were included for the risk estimate without adjustment of age and sex, the analysis was not free of possible confounding factors. ARD and OA are inter-related to each other, and their relationship is a major hurdle to get the pooled estimates of effect size. The size of fabella and its degeneration could not be considered because of variable methods of measurement of dimensions (linear measurement or three-dimensional measurement). Apart from OA and ARD, traumatic injury, chondromalacia, bony stress, physical habits, or occupational need may affect the fabellar prevalence in these studies. But studies included in this meta-analysis did not have sufficient information to stratify for the above factors.

This meta-analysis confirms the role of mechanical stimulus due to knee degeneration and ARD in the prevalence of fabella. The sesamoid bone appears as a cartilaginous nodule, and it is under the regulation of genetic and environmental factors. But, the ossification of fabella in a later stage needs a mechanical stimulus, especially in the form of traction [1]. Sesamoid bone develops in the area of high mechanical stimuli like traction, friction, pressure, and stress. Without mechanical stimuli, genetics, and ARD processes may have a key role in ossification. The genetic influence could not be measured, but the effects of ARD were measured in this meta-analysis. ARD was another predisposing factor that may cause fabellar ossification even in the less active individual. There was a disparity regarding the age of fabellar ossification, but Ehara [20] and Pancoast [26] showed that an ossified fabella might be present as early as 12 years of age. Chung did not discover any cartilaginous fabella in older subjects (>60 years). A proportionate increase in the ratio of ossified fabella and cartilaginous fabella with age was unearthed by Chung [16]. The fabellar prevalence was rising with age and attaining a plateau phase closer to the age of sixty in normal subjects. Laird showed the fabellar prevalence increased with age [27]. A similar finding was also documented by Phukubye and Oyedele [28] but they did not find a correlation with age. Recently, Egerci et al. demonstrated the correlation between fabellar prevalence with age [22]. Conversely, Tabira et al. did not get such an association, which might be due to the low sample size [19].

There were five case reports of fabella syndrome after total knee arthroplasty [25,29]. The mean age of the subjects presenting with fabella syndrome (one male and five females) was 63 years. The case reports showed that ossified fabella often produces clinical symptoms in runners and soccer players [30]. These reports supported the idea of mechanical stimulus or mechanical loading. The gastrocnemius acts as a protagonist at the late stance phase of gait kinematics and undergoes rotational strain during the locking mechanism of knee extension. Popliteus also undergoes rotational strain during the unlocking of the knee. So, the tendons of both gastrocnemius and popliteus develop sesamoids named fabella and cyamella, respectively [1].

The relationship between aging or ARD with fabellar prevalence is controversial. This meta-analysis showed that a moderate relationship existed between both variables. OA was another influencing component and the co-existence of ARD and OA made them covariance for fabellar prevalence. OA was a strong determinant of fabellar prevalence. Hou et al. suggested the fabellar degeneration was correlated with Knee OA [2]. They claimed that the association between OA and fabellar prevalence would be helpful for the diagnosis of existing posterolateral knee pain or post-arthroplasty knee pain or even common fibular nerve palsy. Total knee arthroplasty is the most popular practice for advanced knee OA and its frequency was increasing day by day for a better lifestyle due to the increased life expectancy [25,29]. The majority of subjects are satisfied with the surgery and having pain-free knees, increased knee range of motion, and adequate limb alignment. Some of the patients are troubled with knee pain and orthopaedic surgeon started to search for the potential cause including fabellar disorders [29,30]. The fabellar disorders like impingement or fracture or OA changes may cause postoperative pain and swelling or even catching. The enlarged fabella would impact tibial as well as femoral components and needed excision [4]. The fabellar fracture following arthroplasty may occur due to chronic implant stress or contracture of gastrocnemius muscle for valgus malalignment correction surgery. During preoperative evaluation for knee arthroplasty, a detailed evaluation of posterolateral knee pain and fabellar press test should be conducted [14]. The knee pain could be initiated with irritated and degenerated fabella by applying thumb pressure while moving knee flexion to extension. Sometimes, the common fibular nerve can be compressed during full extension or hyperextension of the knee. A radiological examination must exclude the enlarged fabella (more than 1 cm) with or without OA changes [29]. Intraoperatively, the surgeon must be aware of sclerosed fabellar facet with osteophytes or enlarged fabella inside the lateral head of gastrocnemius tendon. A careful assessment is required while placing an implant regarding fabellar impingement or maintenance of lateral gap following excision of fabella along with fabellofibular ligament.

Limitations of the study

This meta-analysis offers vital information about the fabellar prevalence in OA and ARD with some limitations. The studies included in this meta-analysis did not disclose physical habitus or occupation; hence, the pooled estimates were not free of possible variance. The unequal sample variance (the number of knees per study) may impact the final estimate of the outcome. The small amount of heterogeneity and risk estimates without age-adjustment are some minor limitations due to the unavailability of appropriate data.

## Conclusions

The prevalence of fabella is higher in OA than non-OA subjects and lesser prevalence of fabella in subjects under 40 years. It assists to appreciate the variation of fabellar prevalence in old and young subjects. Coherent with earlier results, the fabellar prevalence is insignificantly higher in the female. The bilateral occurrence of fabella is much higher than the unilateral occurrence. These are related to the distribution of OA, which is bilateral, asymmetrical, and common on the right side. The evaluation of the fabellar prevalence in players, security personnel, and the population residing near the mountains will facilitate understanding of biomechanical stress or load on the fabellar prevalence and the role of fabella in knee kinematics. The stratification of the recommended study will furnish the final estimate of the fabellar prevalence according to physical activity or biomechanical load on the knee.

## Appendices

Search strategy (PubMed):

(((((((Fabella[Title/Abstract]) OR Knee sesamoid[Title/Abstract]) OR Popliteal Sesamoid[Title/Abstract]) OR Sesamoid[Title/Abstract])) AND ((((Prevalence[Title/Abstract]) OR Incidence[Title/Abstract]) OR event rate[Title/Abstract]) OR events[Title/Abstract])) AND (((((Osteoarthritis[Title/Abstract]) OR Knee Degeneration[Title/Abstract]) OR Knee Pain[Title/Abstract]) OR Knee aging[Title/Abstract]) OR Genu pain[Title/Abstract])) NOT Animal').

PROSPERO registration for systematic review and meta-analysis (University of York): CRD42020161834 dated 28/04/2020.
